# Vortex Fluidic Mediated Synthesis of Enhanced Hydrogen Producing Magnetic Gold

**DOI:** 10.1002/smsc.202400449

**Published:** 2025-02-03

**Authors:** Badriah M. Alotaibi, Soraya Rapheima, Po‐Wei Yu, Xianjue Chen, Tanglaw Roman, Christopher T. Gibson, Tiexin Lib, Dechao Chen, Elsa Antunes, Qin Li, Mats R. Anderson, Nadim Darwish, Colin L. Raston

**Affiliations:** ^1^ Flinders Institute for Nanoscale Science and Technology College of Science and Engineering Flinders University Adelaide SA 5042 Australia; ^2^ School of Molecular and Life Sciences Curtin Institute of Functional Molecules and Interfaces Curtin University Bentley WA 6102 Australia; ^3^ School of Environmental and Life Sciences The University of Newcastle Callaghan NSW 2308 Australia; ^4^ Flinders Microscopy and Microanalysis College of Science and Engineering Flinders University Bedford Park SA 5042 Australia; ^5^ Adelaide Microscopy The University of Adelaide Adelaide SA 5000 Australia; ^6^ Environmental Engineering and Queensland Micro and Nanotechnology Centre Griffith University Brisbane QLD 4111 Australia; ^7^ College of Science and Engineering James Cook University Townsville QLD 4811 Australia

**Keywords:** high shear, hydrogen evolution, magnetic gold nanoparticles, photo‐contact electrification, topological fluid flow, vortex fluidics

## Abstract

While bulk gold is well known to be diamagnetic, there is growing experimental and theoretical work supporting the formation of nano gold with unconventional magnetic properties. However, access to such magnetic gold nanoparticles at scale is limited. It is established that magnetic gold particles are readily accessible when exposing aqueous solutions of auric acid (H[AuCl_4_]) to UV irradiation (*λ* = 254 nm) under high shear in a vortex fluidic device (VFD), as a photo‐contact electrification process. Thin films of liquid in the VFD down to ≈200 μm thick are generated in a tilted rapidly rotating angled glass tube with induced mechanical energy imparted under high shear, which when exposed to UV, reduces Au^3+^ to elemental gold without the need for adding reducing agents, unlike in the conventional synthesis of nano gold particles. The use of magnetic force microscopy (MFM) is reported to show that VFD‐generated 2D gold sheets have magnetic gold nanoparticles embedded in them, with the material electron paramagnetic resonance active. A report is made on theoretical insights into the origin of the magnetism and that the material shows a dramatic enhancement of catalytic activity in the hydrogen generation reaction relative to using traditionally produced gold nanoparticles of comparable size.

## Graphical abstract

Magnetic gold particles embedded in 2D gold sheets are readily formed when exposing aqueous solutions of auric acid (H[AuCl_4_]) to UV, *λ* = 254 nm, under high shear in a vortex fluidic device, as a photo‐contact electrification process, with the electron paramagnetic resonance active material dramatically enhancing catalytic activity in the hydrogen generation reaction.

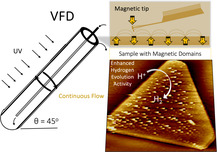

## 1 Introduction

Anthropogenic climate change is being accelerated due to increasing uses of fossil fuels, and there is now growing attention to the development of sustainable energy, including hydro, wind, and solar sources, to ultimately circumvent any greenhouse gas emissions. However, the efficiency of these sustainable energy sources varies according to location, weather, season, and time, requiring them to be combined with energy carriers for the purpose of energy storage or shipping. Hydrogen has emerged as a promising and competitive candidate due to its high energy density (MJ/kg) and the extremely clean exhaust.^[^ ^1^ ^]^ To produce green hydrogen the electrochemical hydrogen evolution reaction (HER) has been considered as a promising and effective method.^[^ ^2^, ^3^ ^]^ Because of the high kinetic barrier of water splitting, electrocatalysts are required to promote hydrogen conversion efficiency, and for this purpose gold (Au)‐based catalysts offer good efficiency.^[^ ^4^ ^]^ Considering the high cost and limited reserves of Au metal, the development of efficient approaches to reduce its downsizing to nano‐size domains for increased area of active site while improving its catalytic performance is essential. The design of advanced Au‐modified electrocatalysts based on nanostructure has already achieved significant progress in water splitting^[^ ^4^, ^5^, ^6^ ^]^ owing to their distinctive optical and chemical characteristics.^[^ ^7^, ^8^ ^]^ We note that magnetic gold particles have not been used in water splitting, possibly because of the limited scalability of making such material, and this is explored in the present study.

There are several experimental and theoretical studies focused on magnetic properties of gold at the nanoscale, even though elemental gold is generally known as a diamagnetic material in its bulk structure.^[^ ^9^, ^10^, ^11^, ^12^ ^]^ A single atom of gold has an odd number of electrons, so it has one unpaired electron. But in bulk, these unpaired electrons can be shared between atoms, allowing them to form a pair of electrons. Hence, the band structure of gold and its density of states show a balanced number of spin‐up and down electrons, and it is therefore diamagnetic^[^ ^13^, ^14^, ^15^ ^]^ and devoid of classical magnetism. However, there are several reports about magnetic gold at the nanoscale, specifically between gold nanoparticles (AuNPs) dimers with the two unpaired electrons not paired, and the dimer is consequentially magnetic. The magnetic susceptibility of AuNPs varies greatly from bulk gold, depending on their size, shape, and surface effects. At the nanoscale, surface and volume effects have an impact on magnetic phenomena, which change the electronic structure of materials. Charge carriers can flow continuously across energy states in bulk metals, but in nanoparticles there is a transfer toward discrete energy states, caused by quantum confinement effects.^[^ ^16^ ^]^ Because of the geometrically confinement of electrons and the high percentage of surface atoms in nanoparticles, new magnetic characteristics arise. As a result of energy minimization, surface charges may be transported to the interior of the material, and unbalanced charge spins may emerge close to the surface of nanoparticles. The unbalanced spins can cause additional magnetic moments.^[^ ^17^ ^]^ Magnetic properties of AuNPs, nanoclusters, and nanocrystalline films have been reported^[^ ^18^, ^19^, ^20^, ^21^, ^22^ ^]^ with the magnetism depending on the size of the nanoparticles, the way they are made and their surface chemistry.^[^ ^11^, ^12^, ^23^ ^]^ However, there is no clear understanding on the origin of the magnetism. Most approaches reporting the formation of magnetic nano gold involve the use of various surface stabilizer molecules, for example, where small AuNPs are coated with thiol groups,^[^ ^24^ ^]^ with such surface modification playing a crucial role in altering the magnetic properties of the particles. In general, the rearrangement of charge carriers due to surface modifications can result in magnetic behavior of gold particles.^[^ ^25^ ^]^ While consensus exists, that magnetism in AuNPs is induced by surface effects, the specific role of capping ligands remains a subject of ongoing study. Compelling evidence suggests that magnetism is modulated through changes in the electronic properties resulting from the interaction of surface gold atoms with the organic ligands, which provides insights into the reversible modulation of magnetic saturation in AuNPs.^[^ ^25^ ^]^

In this article, we study the magnetic response of pure AuNPs prepared under continuous flow in the vortex fluidic device (VFD) using water as benign solvent in the absent of hazardous chemicals and surfactants, the continuous flow method addressing scalability of any process at its inception. The VFD is a versatile thin‐film microfluidic platform with a growing number of applications.^[^ ^26^, ^27^ ^]^ The thin film of liquid in the VFD has complex fluid dynamics, beyond the expected Stewartson/Ekman layers, encompassing high shear topological fluid flows of submicron diameters, as spinning top (typhoon‐like) flow as a Coriolis force from the curved base of the tube, and double helical flow, as twisted eddies arising from Faraday waves and side wall Coriolis force. Under continuous flow for the rapidly rotating tube titled at 45°, which is the optimized angle for processing in the device, the liquid moves up the against gravity, exiting at the top of the tube.^[^ ^28^ ^]^

We have recently reported a detailed understanding on the synthesis of AuNPs directly from aqueous auric acid (H[AuCl_4_]), without the need for adding a reducing agent or surfactant, in a thin film of liquid in the VFD when irradiated with UV at *λ* = 254 nm.^[^ ^29^ ^]^ The preferential formation of different AuNPs in the VFD depends on the different high shear topological fluid flows in the rotating quartz tube. The VFD processing allows access to ultrathin 2D sheets, prisms, hierarchical structures comprised of AuNPs embedded within these sheets, spheroidal particles, and rosette and tubular structures.^[^ ^29^ ^]^ The formation of gold particles is primarily from photo‐contact electrification (photo‐CE) with their surfaces devoid of capping agents, unlike in conventional batch processing. The photo‐CE occurs at the solid (tube surface)–liquid interface with the oxidation of water photo‐induced, forming the hydroxyl radical, OH^•^. In air the redox couple is reduction of ^3^O_2_ to the superoxide radical anion, O_2_^−•^, which then reduces Au^3+^ to elemental gold, as does other reactive oxygen species present, competing with photo‐CE reduction of Au^3+^ on the surface of the tube and coupled with the photo‐induced oxidation of water.^[^ ^29^ ^]^

We find that AuNPs prepared in the VFD is magnetic, which is studied herein using magnetic force microscopy (MFM). With now the ability to make magnetic AuNPs we sort to explore their potential in the HER and find that it is more effective than conventionally prepared nonmagnetic AuNPs (see below). The ability to readily prepare magnetic AuNPs further highlights the utility of the VFD in gaining access to functional nanomaterials beyond what is possible using traditional processing strategies. Indeed, surfactant free morphologically disparate 2D gold sheets and spheroidal magnetic gold nanoparticles incorporated as catalysts in the hydrogen generation reaction are more effective catalysts as such than traditionally produced AuNPs. This finding also provides insight into the utility of the VFD in fabricating novel materials with beneficial green chemistry metrics both for their synthesis and applications.

MFM is a powerful characterization technique, originally developed as a derivative of the atomic force microscopy (AFM). MFM has the ability for imaging and detecting magnetic interactions between a magnetized tip and nanomaterials deposited on a substrate under both ambient and buffer conditions.^[^ ^30^, ^31^ ^]^ Essentially, MFM can distinguish the magnetic and nonmagnetic materials at the micro‐ and nanoscale dimensions.^[^ ^32^ ^]^ It is used extensively in studying and localizing magnetic domains on substrates such as magnetic bubble^[^ ^33^ ^]^ magnetic vortices in nanodisks^[^ ^34^ ^]^ and skyrmions in helimagnets.^[^ ^35^, ^36^ ^]^ MFM was used herein to characterize the magnetic response by analyzing the phase shifts with a negative phase shift establishing an attractive interaction between the magnetic tip and the AuNPs. The magnetic behavior is also supported by responsive electron paramagnetic spectroscopy (EPR) studies. Given the experimental evidence for magnetic gold and the potential of such material in different applications, a comprehensive understanding of the magnetic behavior for various gold systems has been further sought using density functional theory (DFT).

## 2 Results and Discussion

**Figure 1:**
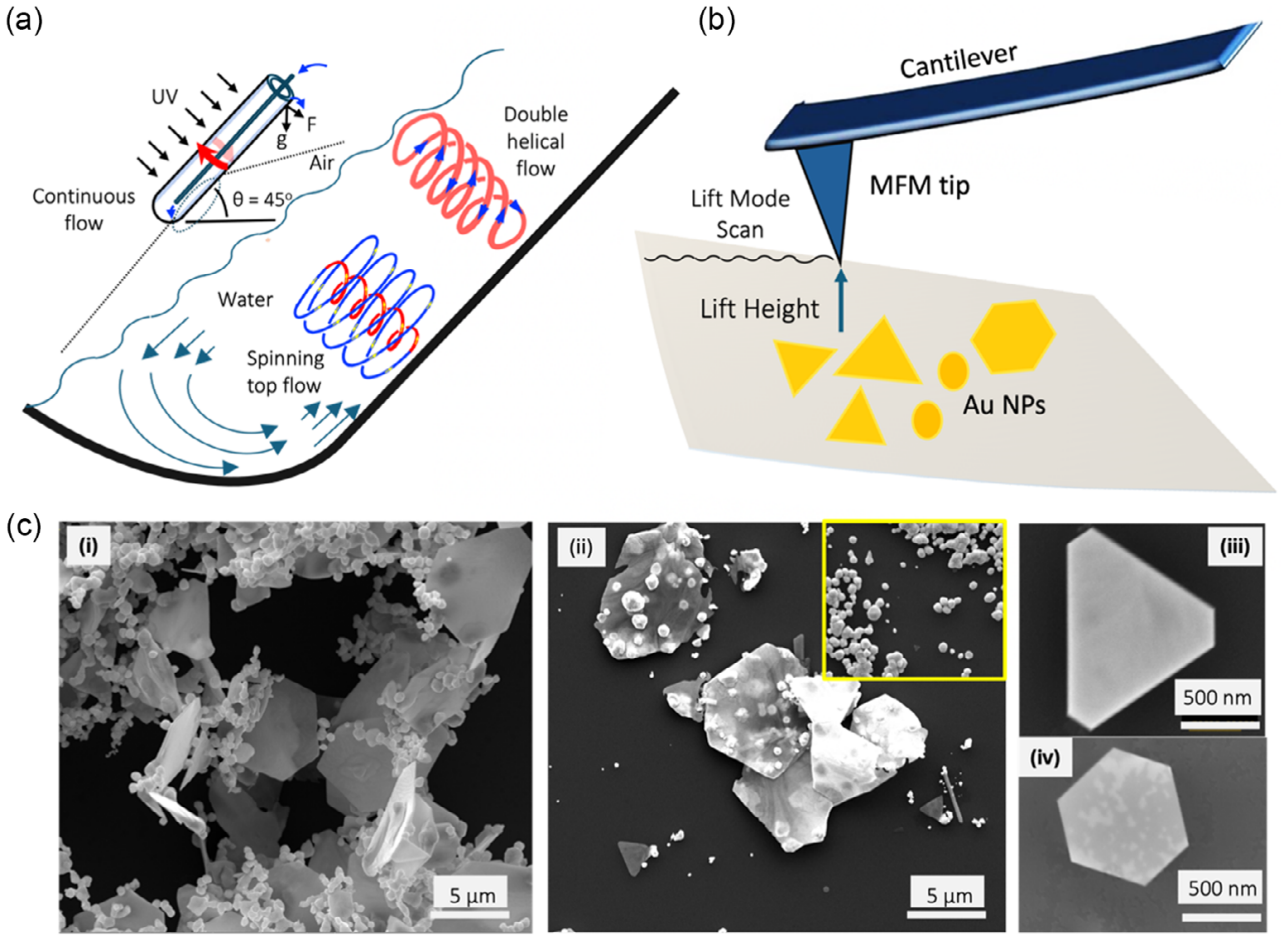
a) Schematic of the vortex fluidic device (VFD) showing the typhoon‐like spinning top (ST) topological fluid flow from the Coriolis force from the hemispherical base of the rapidly rotating tube, and double helical flow from the induced Faraday wave eddies coupled with the Coriolis force from the curve surface along the tube. b) Schematic illustration of the magnetic force microscopy (MFM) setup. The probe in the MFM is coated with a magnetic material and the cantilever supports the probe at one end allowing for precise movement and force measurements. c) SEM images of VFD prepared ultrathin 2D triangular and hexagonal gold sheets with small nanoparticle on the surface of the sheets; VFD processing was as follows: *ω* = 5 k rpm, *θ* = 45°, *λ* = 254 nm, *c* = 3.7 mm, under air atmosphere.

High shear regimes in water in the VFD result in situ reduction of gold under UV irradiation as a photo‐CE processing at the liquid–solid interface.^[^ ^29^ ^]^ Wang et al. have established contact electrification processes in water^[^ ^37^ ^]^ with electron transfer as the dominant mechanism occurring at liquid–gas, liquid–liquid, and solid–liquid interfaces.^[^ ^37^, ^38^ ^]^ In all liquids, an electrical double layer is formed between liquids and solids, which is the primary surface for CE. This phenomenon in the VFD relates to the presence of localized high temperatures and high pressures within the topological fluid flows. Herein the AuNPs were prepared using a variance of our recently reported method involving UV irradiating thin a film of aqueous auric acid within the rapidly rotating VFD quartz tube tilted at 45°, **Figure** **1**a.^[^ ^22^ ^]^ Water is oxidized at the solid–liquid interface, forming the hydroxyl radical OH^•^. Redox couples occur in air when ^3^O_2_ is reduced to superoxide radicals, such as O_2_^−•^, which in turn reduces Au^3+^ to elemental gold,^[^ ^29^ ^]^ as does other reactive oxygen species present, along with CE reduction of Au^3+^ on the tube surface, followed by photo‐induced water oxidation. Based on the VFD processing parameters, the nano gold structures can be isolated as ultrathin 2D sheets (typically 5–10 nm thick), prisms, hierarchical structures containing nanoparticles embedded in these sheets, and rosette and tubular structures.^[^ ^29^ ^]^ Scaling up the process resulted in ≈60–70% yield with a flow rate of 0.3 mL min^−1^.

### 2.1 Magnetic Force Microscopy (MFM)

**Figure 2:**
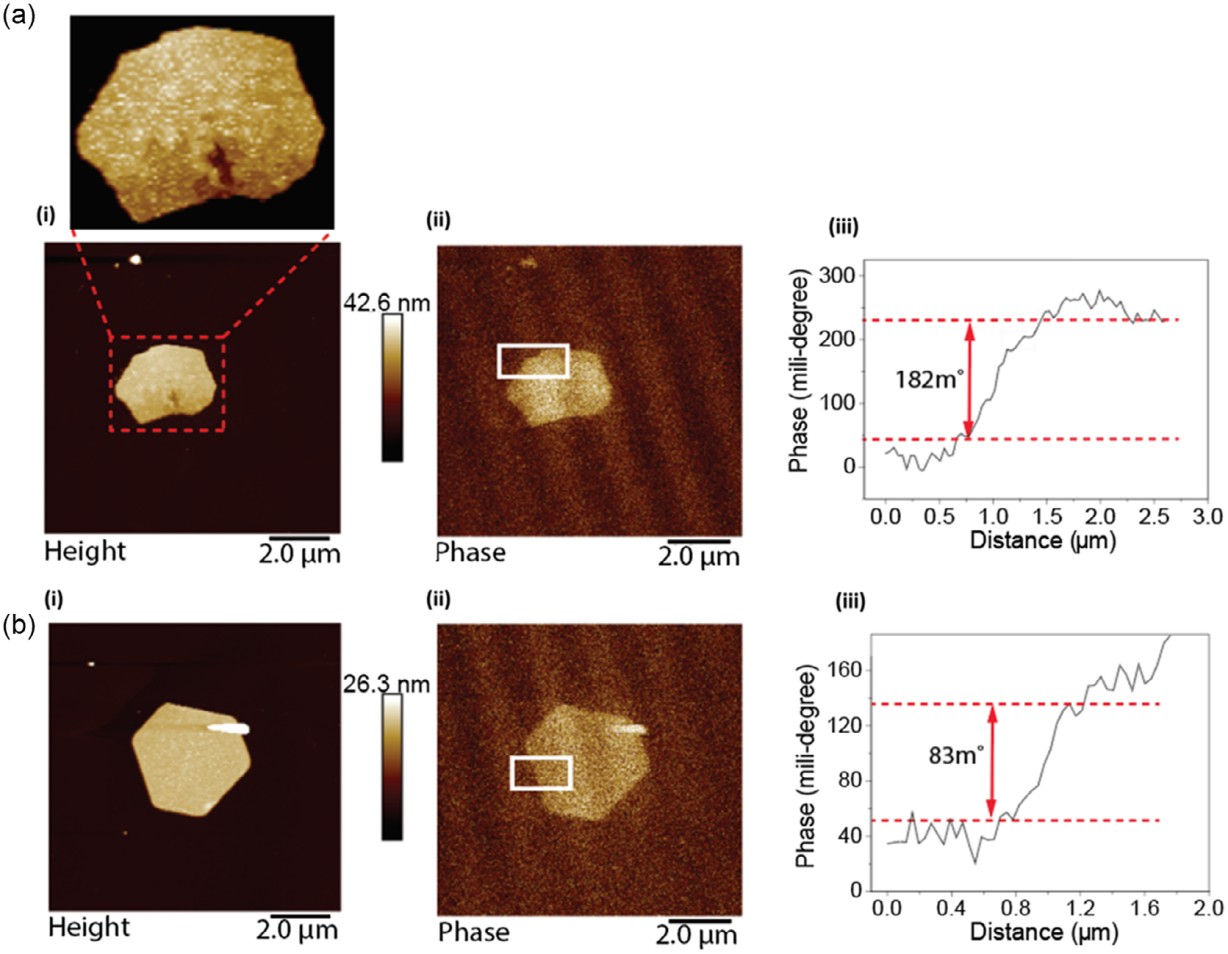
a) (i) MFM topography, (ii) MFM phase image, and (iii) the corresponding phase shift profile of the rectangle in (ii), respectively. Inset in (a) clearly shows the presence of gold nanoparticles inside the 2D gold sheet, b) (i) MFM topography image, (ii) MFM phase image for different sheets location and (ii) the corresponding phase shift profile of the rectangle in (ii), respectively. The lift scan height is 50 nm. VFD processing was as follows: *ω* = 5 k rpm, concentration of auric acid = 3.7 mM and *θ* = 45°, 254 nm UV light, air atmosphere, under continuous flow with a flow rate 0.3 mL min^−1^.

The properties of the Au 2D sheets, spherical nanoparticles and tubular structures has been recently published.^[^ ^29^ ^]^ Herein, we focus on the magnetic properties using MFM, with each line of MFM image measured twice, one for sample topography and the second for measurements of magnetic fields at a fixed probe–sample distance.^[^ ^39^ ^]^ First, measurements were performed on the 2D gold sheets that were formed under confined and continuous flow modes of processing in the VFD, which showed magnetic responses for both 2D triangles and hexagonal sheets with positive phase shifts in the phase shift profiles, **Figure** **2**.

The phase shift ranges from 80 to 900 millidegrees, Figure 2a,b(iii), or from 600 to 900 milli degrees, Figure S1 (Supporting Information), for different sheets and different areas. This can be attributed to different thicknesses of the 2D sheets and different accumulation of nanoparticles within them. A positive phase shift means that the magnetic moment of the nanoparticles is oriented opposite to the magnetic field of the tip. The lift heights of 30, 50 and 100 nm were chosen because these are typical lift heights used in MFM.^[^ ^40^, ^41^ ^]^ At lift heights above 100 nm, the signal becomes too weak to reliably detect the magnetic features, making it challenging to obtain accurate measurements. Conversely, at lift heights below 30 nm, there is significant interference from the surface topography, which can distort the magnetic signal. These optimal lift heights balance signal strength and resolution, ensuring reliable data acquisition.

**Figure 3:**
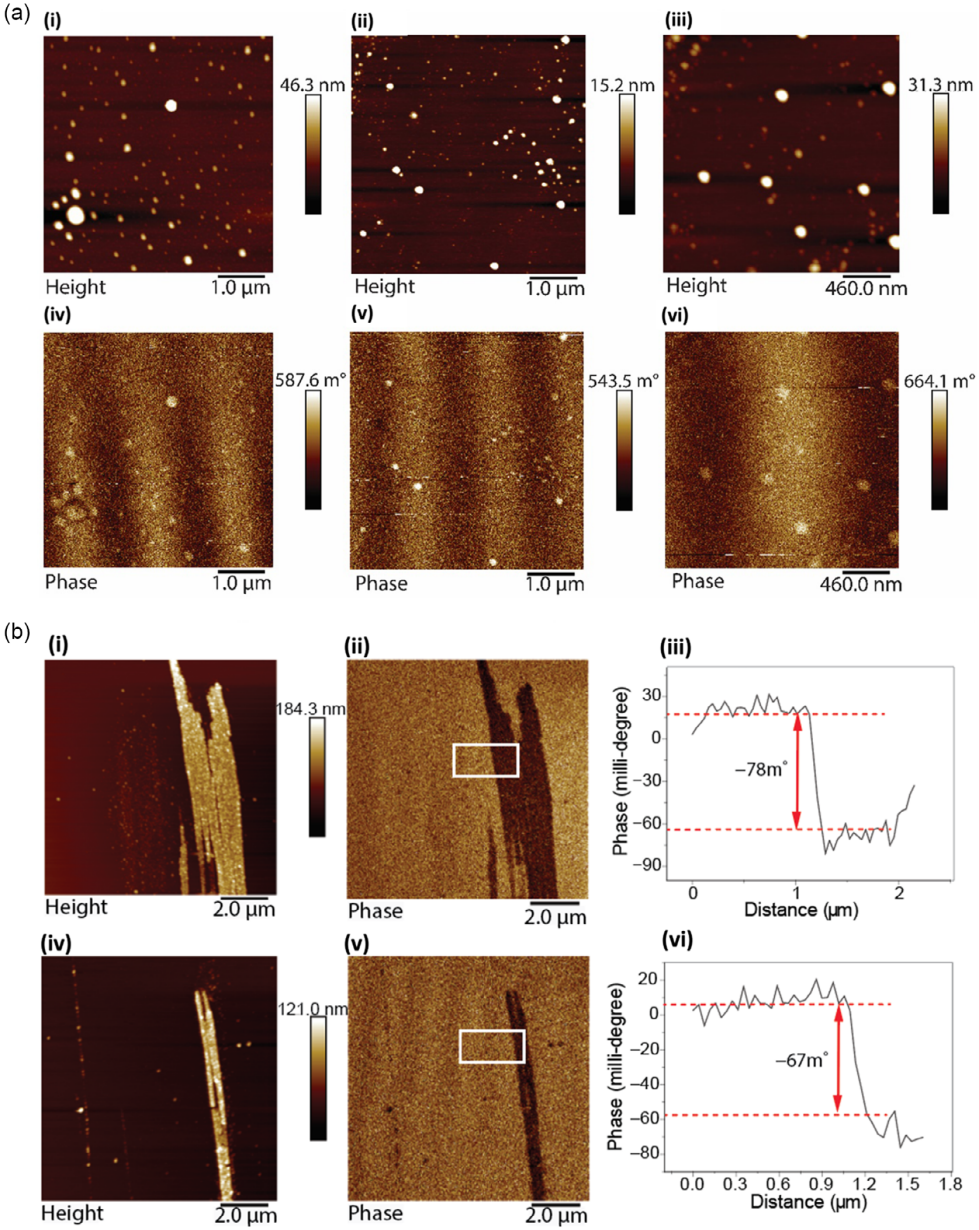
a) MFM measurements on Au spherical nanoparticles. (i)–(iii) MFM height topography images. (iv)–(vi) MFM phase images indicating positive phase shift. The lift scan height is 50 nm. VFD processing was as follows: *ω* = 5 k rpm, *θ* = 45°, *λ* = 254 nm, *c* = 3.7 mM, in air. b) (i) MFM topography on Au tube structure, (ii) MFM phase image and (iii) the corresponding phase shift profile of the rectangle in (ii), respectively. (iv)–(vi) MFM topography image MFM phase image and the corresponding phase shift profile of the rectangle in (v), respectively. The lift scan height is 100 nm. VFD processing was as follows: *ω* = 5 k rpm, *θ* = 45°, *λ* = 254 nm, *c* = 3 mM, air atmosphere under continuous flow with a flow rate 0.3 mL min^−1^.

The sheets have small nanoparticles on their surfaces which are the centers of the magnetic response. This was also consistent with MFM measurements on spherical nanoparticles fabricated using the VFD, **Figure** **3**a, which show a degree of magnetism, as evidenced by a positive phase shift in the MFM phase images, Figure 3a. Thus, the small nanoparticles are themselves magnetic, and it is reasonable to suggest that the magnetism in the 2D sheets originate from the magnetism of the nanoparticles themselves.

The tube structure for which their synthesis has been reported before^[^ ^29^ ^]^ was also studied using MFM, noting that both the 2D sheets and the tube have potential to encapsulate nanoparticles within their structures, and is highlighted in Figure 3b. The MFM results also showed magnetic responses for pod structures with negative phase shifts in the phase shift profiles, as shown in Figure 3b. The reason why the magnetic dipoles for the 2D sheets are opposite to that of the tube structure (positive versus negative phase shifts) with respect to the magnetic field of the tip remains unclear. We suggest that the opposite dipole moments are due to the different stacking of the nanoparticles in the 2D and tubular structures. Mainly 2D gold is formed in high yield in the VFD when the inner surface of the tube is coated with a layer of silica xerogel^[^ ^29^ ^]^ and it also has a magnetic response, Figure S2 (Supporting Information). Moreover, MFM measurements on the 2D gold material formed under an inert atmosphere of nitrogen in the VFD,^[^ ^29^ ^]^ using a Young's tap modified VFD tube, also showed a magnetic response, Figure S3 (Supporting Information), and thus the magnetic properties of the gold formed in the VFD is not originating from oxygen‐induced radical species.

### 2.2 The Origin of the Magnetism

To gain further insight into the nature of the novel gold materials, electronic ground state studies on the lowest‐energy structures using Kohn–Sham density functional theory (DFT), and EPR studies along with transmission electron microscopy (TEM) studies were undertaken.

**Figure 4:**
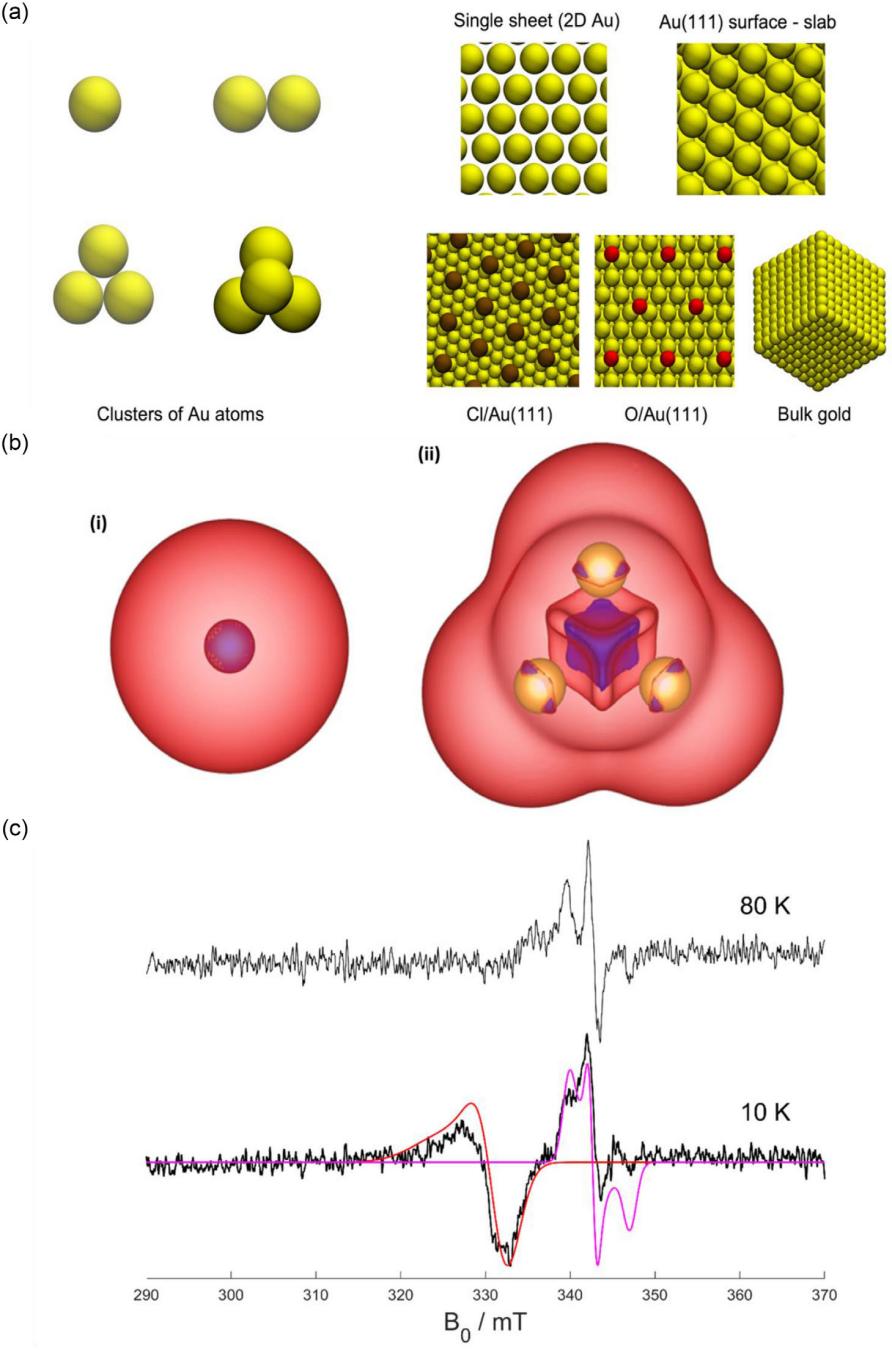
a) Atomic systems of gold studied with DFT calculations. The ground electronic states of the four small clusters of Au atoms to the left of the figure were found to be magnetic, while the five extended systems of gold (pristine and with adsorbates) were found to be nonmagnetic. b) Spin density distribution of electrons on (i) a single Au atom, and (ii) on a cluster of four Au atoms. Spin‐up density is shown in red, and spin‐down in blue. The total magnetizations in the systems are (i) 1.00 $\mu_{B}$, and (ii) 2.03 $\mu_{B}$. c) X‐band (9.6139 GHz) CW EPR spectrum recorded at 10 and 80 K of AuNPs under air in water with 10% v/v glycerol. Blue – experiment, simulation red (*g* = [2.064 2.064 2.110], linewidth = [120 120 250] MHz), magenta (*g* = [2.012 1.996 1.970], linewidth = [50 30 50] MHz).

Electronic ground states and the lowest‐energy structures of different gold systems were determined using DFT as implemented in the Vienna Ab initio Simulation Package (VASP)^[^ ^11^, ^12^ ^]^ version 6.3.1. The DFT calculations aim to determine the origin of the magnetism for gold particles prepared in the VFD. It seeks to elucidate the origin of magnetism exhibited by these particles by probing the electronic structure and interactions within the gold particles. These systems include: 1) a free‐standing gold atom, 2) a gold dimer, 3) a gold trimer, 4) a gold tetramer, 5) a sheet of gold (single‐atom thickness), 6) a slab of gold approximating Au(111), 7) bulk gold, 8) Au(111) with 1/9 monolayer (ML) chlorine, and 9) Au(111) with 1/9 ML oxygen, **Figure** **4**a. Results show that small clusters of gold atoms exhibit magnetic character. Figure 4b plots the magnetization in small gold clusters, showing a more prominent spin‐up electron distribution in the 1‐ and 4‐atom clusters that yield a non‐zero magnetic character. This magnetization is not present in the more extended systems considered: sheets, surfaces, and bulk gold. Adding Cl and O adsorbates on gold surfaces does not make the gold surface magnetic, noting that there are no caping agents on the magnetic gold prepared herein.

TEM images were taken on the small Au NPs and the 2D sheets, Figure S4 (Supporting Information), showing the presence of both gold nanosheets and small clusters as large as 200 nm in diameter, which is consistent with the SEM results. The selected area electron diffraction pattern, Figure S4 (Supporting Information), of a typical gold nanosheet shows hexagonal symmetry. This is an indication that the platelet is highly <111> oriented with the top normal to the electron beam, being indexed as the face‐centered‐cubic (*fcc*) structure of elemental gold. The zoomed‐in TEM image and the corresponding fast Fourier transform (FFT) pattern of the lattice image in Figure S shows a typical ring‐like pattern corresponding to the (111), (200), and (220) reflections from the gold. From TEM analysis, it is evident that small clusters are present, which could potentially contribute to the observed magnetism.

Previous studies have shown that the maximum size of AuNPs with detectable magnetization is no larger than 27 nm.^[^ ^17^ ^]^ The 100 nm size of the magnetic AuNPs observed here appear to be an abnormality. Electron paramagnetic resonance (EPR) spectroscopy is applied to understand further the origin of the magnetism.^[^ ^42^ ^]^ The two EPR signals shown in Figure 4c suggests that there exist two kinds of defects or unpaired electron states in the colloidal AuNP suspension. They are likely caused by structural defects in the AuNPs, noting that they are formed under high shear vortex field under which complete surface energy minimization is likely unachievable, leaving point defects or misalignment on crystal facets. These defects may attract H_3_O^+^, subsequently OH^−^, rendering negative charge to the AuNPs and making them a suspension. The EPR signals obtained in the present work don't fit that for atomically precise gold nanoclusters,^[^ ^43^ ^]^ which are formed in the absence of high shear stress and are much smaller than our AuNPs. Defects have been identified as the origin of the magnetism in these nanoparticles which are formed under high shear in a quartz VFD tube and the presence of EPR active transition metal contaminants is unlikely.

To investigate whether the magnetism is due to how the nanoparticles are generated in the VFD, we performed MFM measurements on pre‐prepared AuNPs using ascorbic acid (classic gold batch processing) prior to the VFD processing as well as pre‐prepared AuNPs with ascorbic acid introduced into the VFD. The nanoparticles with ascorbic acid were prepared by mixing 1:1 ascorbic acid (AA) solution (concentration of AA, 1 mg in 1 mL of water), with 3.7 mm of gold chloride solution and then stirred for 10 min. The results show no magnetic response for such AuNPs prepared using ascorbic acid with and without using VFD indicating that the magnetic properties of the AuNPs and the 2D sheets arise from nucleation process of the nanoparticles in the VFD, as shown in Figure S5 and S6 (Supporting Information).

To determine that the magnetic response is not due to electrostatic effects, MFM measurements were also performed on the VFD‐fabricated 2D gold sheets with bias of different polarities applied to the sample. Applying the bias effectively cancels electrostatic forces and consequently shadows the magnetic properties of the 2D sheets in case the obtained magnetic profile is due to the electrostatic effects. This means that only the magnetic sheets with real magnetic properties will show significant phase shifts in the MFM phase images. The control experiments showed that the intensity of the magnetism for 2D gold sheet remained constant after applying a bias of ±1000 mV, confirming that the magnetic response arises from the nature of 2D gold sheets after processing in the VFD with no contribution of electrostatic forces, as shown in Figure S7a (Supporting Information).

The observed magnetism is intrinsic to the nanoparticles themselves, rather than being caused by external factors such as the presence of the presence of oxygen inside the gold lattice or defects on the surface of the nanoparticles. We used here more analytical techniques such as thermal gravimetric analysis (TGA) and Raman spectroscopy, which can characterize the presence of oxygen in the gold lattice. There is no peak in the Raman spectrum corresponding to any oxygen trapped in the gold, Figure S7d (Supporting Information), reflecting that it is a normal gold surface, and it is consistent with magnetic response present when forming the gold material under an atmosphere of nitrogen, Figure S3 (Supporting Information). It is also consistent with TGA results, there being no evidence for weight loss on heating, Figure S7b (Supporting Information). MFM measurements were also performed for the fabricated 2D gold sheets within the VFD after heating them to 900 °C, which is below the melting point of bulk gold, at 1063 °C. The results showed the same magnetic behavior for the sheets before and after thermal processing, Figure S7c (Supporting Information).

In the present study, we have determined that the magnetic response observed in AuNPs processed in the VFD is intrinsic to the nanoparticles themselves. We have studied several factors in understanding the origin of the gold magnetism, including the presence of oxygen in the gold lattice, surface defects, or external factor such electrostatic forces. The magnetic properties are inherent to the nanoparticles formed during nucleation and growth in the VFD. SEM and TEM analysis revealed the formation of small gold clusters, alongside nanosheets. This structural information supports the hypothesis that small gold clusters are key contributors to the observed magnetism, as confirmed by the DFT simulations, which indicate that small gold clusters exhibit magnetic characteristics. There are no defects observed using TEM, but EPR signals detected suggest the presence of structural defects or unpaired electron states in the Au NPs, likely induced by the high shear vortex field, which prevents complete surface energy minimization and leaves defects or misaligned crystal facets. Additionally, the magnetic response was consistently observed in the 2D gold sheets formed in an inert nitrogen atmosphere, ruling out the influence of oxygen‐induced radicals. This is consistent with the lack of any oxygen‐related peaks in the Raman spectrum, as well as the absence of weight loss in TGA analysis, further confirming that oxygen‐induced species are not responsible for the magnetic response. MFM measurements performed after heating the gold sheets to 900 °C showed no change in magnetic behavior, indicating that the observed magnetism is stable and intrinsic to the material, rather than being affected by the surrounding environment or thermal treatment. Overall, the magnetic response of the AuNPs is dependent on a combination of several factors, namely (a) the electronic structure and (b) the effect of defects and (c) the nucleation process that occurs under the specific conditions within the VFD. Previous theoretical reports describe a surface effect with an important orbital contribution to the magnetic moment.^[^ ^44^, ^45^ ^]^

### 2.3 Hydrogen Evolution Reaction (HER)

**Figure 5:**
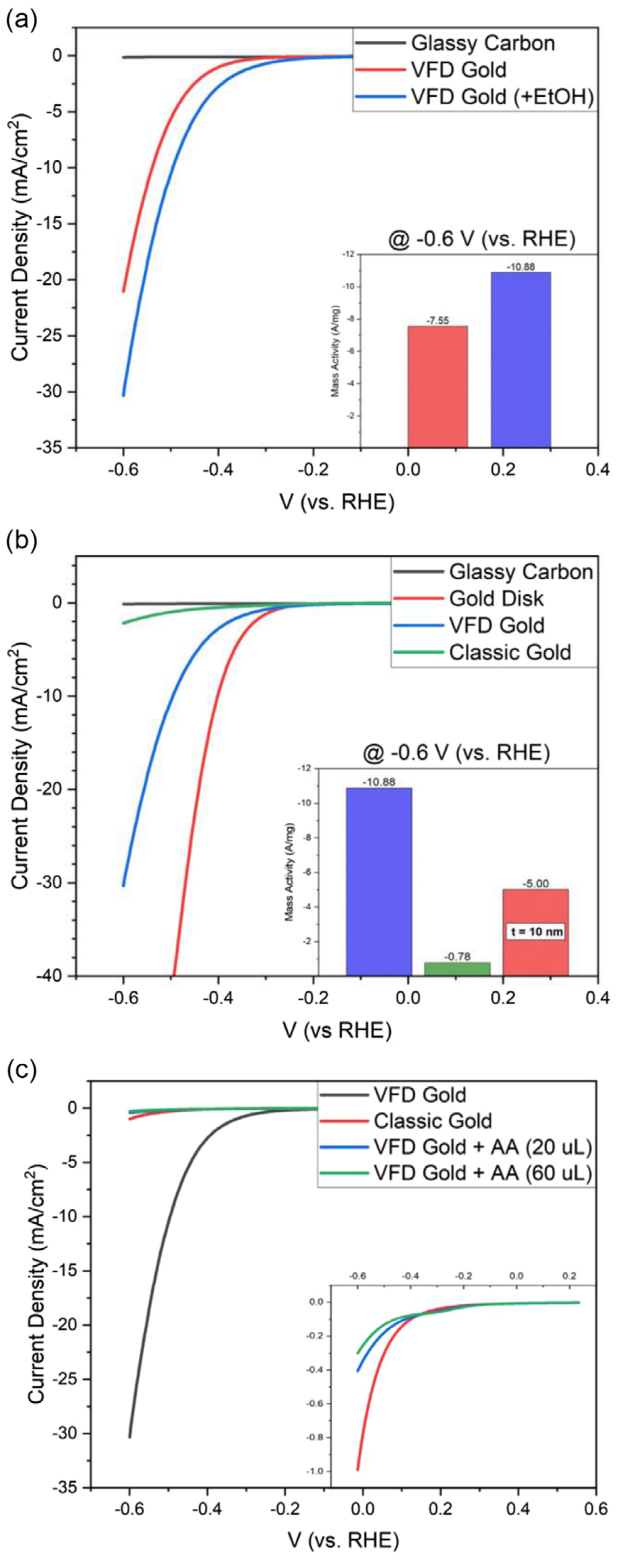
The forward scan of cyclic voltammogram sweeping between 0.235 to −0.6 V (vs RHE) presenting HER activity in 0.5 M H_2_SO_4_ from a) VFD‐generated magnetic nano gold dispersed in 50 vol% ethanol/water. b) Magnetic gold in comparison with classic gold and gold disk benchmark catalyst. The calculated mass activity is inserted here, and in the graph shown in Figure S8 (Supporting Information). c) Magnetic gold before/after adding ascorbic acid (AA) solutions compared to classic gold combined with an inserted figure zooming in the current density range between 0.1 and −1.1 mA cm^−2^. VFD processing was as follows: *ω* = 5 k rpm, *θ* = 45°, *λ* = 254 nm, confined mode, under air atmosphere.

Here, we investigate the activity of the aforementioned magnetic AuNPs mixture in the HER. As what has been mentioned, the AuNP dispersions were prepared in water which would cause the “coffee‐ring” effect whereby particles agglomerate while drying the electrode. This results in the accumulation of AuNPs, rendering buried AuNPs inactive in HER. To minimize the “coffee‐ring” effect and provide improved separation of AuNPs on the electrode surface, the 1.5 mM gold nanoparticle dispersions were diluted to a target concentration of 0.2 mM, Figure S8 (Supporting Information), with both water and ethanol to achieve the dispersion of gold nanoparticles in 50 vol% ethanol/water.^[^ ^46^ ^]^ **Figure** **5** a presents the comparison of current density between magnetic gold prepared in the VFD dispersed in pure water and 50 vol% ethanol/water. The addition of ethanol to a magnetic gold dispersion can significantly boost the HER activity by ≈50% at –0.6 V (vs RHE) with a lower onset potential. Due to its enhanced HER activity, a 0.2 mm magnetic AuNPs dispersion in 50 vol% ethanol/water was used in the next comparison with benchmark catalyst and nonmagnetic gold (classic batch gold), Figure 5b.

We conducted a comparative study on the catalytic activity of magnetic gold versus nonmagnetic (AA) alongside gold disks. This was to emphasize key indicators of catalytic activity given the challenge of effectively comparing and evaluating the performance of materials due to variations in reported methods in literature.

Undoubtedly, the pure gold disk electrode in which the whole surface can be used to catalyze the HER outperforms magnetic gold prepared in the VFD, owing to the significant difference in surface coverage of gold. Nevertheless, when normalizing the HER efficiency with the amount of gold used on the electrodes, the magnetic AuNPs present a better mass activity (A/mg). For the comparative analysis, the thickness of gold disk electrode was set to 5 nm to mimic gold nanofilm which is extremely thin in comparison with magnetic AuNPs (average size of gold nanoparticles 100 nm.^[^ ^29^ ^]^) Under this condition, the HER performance of magnetic gold nanoparticles is double that of the 5 nm gold film benchmark, highlighting its excellent metal utilization efficiency. As for another comparison, a poor HER performance was observed for the classic AuNPs, which might arise from ascorbic acid acting as a caping agent, thereby insulating the classic gold.

In order to identify whether the ascorbic acid (AA) in classic gold dispersions is the main reason of low HER activity, two different volumes (20 and 60 μL) of ascorbic acid solution (1 mg of AA in 1 mL of water) was added into 200 μL magnetic gold dispersion which exhibited a good HER activity, Figure 5b. After adding the ascorbic acid solution, the aggregation of gold nanoparticles was triggered. The HER activity of the ascorbic acid added magnetic gold dispersions drop from −30 mA cm^−2^ to less than −1 mA cm^−2^ at −0.6 V (vs RHE) which is even less efficient than classic gold thereby proving that the existence of ascorbic acid in magnetic gold dispersion causes a reduction in HER activity, Figure 5c. The impact of magnetic properties of the electrode in the HER has been reported.^[^ ^47^ ^]^ Owing to the unpaired electron spins on magnetic gold nanoparticle, the interfacial magnetic gradient leads to enhanced electron transfer, promoting the HER efficiency. When cooperating with Nafion, the HER activity significantly increases. The synthetic method reported herein involving photo‐CE in the VFD avoids the need of the addition of ascorbic acid while maintaining magnetic behavior, making it more ideal for the electrochemical applications.

## 3 Conclusion

The findings establish a paradigm for VFD processing in water under UV irradiation involving photo‐induced CE, which allows access to different magnetic nano gold material beyond what is possible using traditional batch processing strategies, with the surfaces pristine, being devoid of auxiliary agents, such as surfactants. This also makes the process attractive in terms of green chemistry metrics, as well as avoiding the effect of surface species on potential applications. MFM established that there is magnetic interaction for the gold, presumably arising from more efficient packing within the gold with more efficient tunneling of electrons, with the material having an EPR response. The origin of the magnetism for the AuNPs is dependent on electronic structure, the effects of defects and the mechanism of their nucleation and growth within the VFD. The preparation of pristine magnetic AuNPs makes them more ideal for the electrochemistry and in catalyst HER in different ways, being more effective than conventionally prepared nonmagnetic AuNPs. Given that the magnetic AuNPs can be stably dispersed in 50% v/v ethanol in water, AuNP dispersions can be spray‐coated onto larger electrode surfaces efficiently for large‐scale hydrogen generation, noting that the gold nanoparticles can be prepared at scale in the VFD. The findings highlight the utility of the VFD in gaining access to functional nanomaterials beyond what is possible using traditional processing strategies.

## 4 Experimental Section

4.1

4.1.1

##### Materials

Auric acid (H[AuCl_4_]), 99.9% purity in aqueous HC, and ascorbic acid were purchased from Sigma Aldrich. Ethanol was purchased from Chem‐Supply. Milli‐Q water was used for all the processing.

##### Synthesis of Gold Nanoparticles (VFD Generated)

Methods of nano gold preparation was adapted from reference.^[^ ^29^ ^]^

The as‐received aqueous auric acid (H[AuCl_4_]) was diluted in Milli‐Q water to obtain 3.7 and 3.0 mm concentrations. The auric acid solution was used directly in a VFD operating at room temperature under air unless otherwise stated. For the confined mode of operation of the VFD, 1 mL of the diluted solution was placed in a 20 mm outside diameter (OD) (17.5 mm internal diameter (ID)) quartz tube, 18.5 cm in length, with a hemispherical base and open at the other end, essentially taking on the shape of a conventional test tube. The tilt angle (*θ*) of the tube was fixed at 45° unless otherwise stated with the tube spun at the specified rotational speed (*ω*), for a designated time. For continuous flow processing the tube was also tilted at 45° with a flow rate of the same solutions at 0.3 mL min^−1^ unless otherwise stated. For all processing in the VFD, the quartz tube was irradiated on both sides along the length of the tube, with UV‐LEDs operating at approximately *λ* = 254 nm, 20 W, for 60 min processing in the confined mode and for a certain volume of liquid, as specified, or under continuous flow at the above flow rate. The resulting solutions were centrifuged for 1 min at 6900 × g whereupon the resulting gold material was washed with Milli Q‐water (1 mL). Samples were then drop casted onto silicon wafers and left to dry in air for characterization purposes.

##### Characterizations: Scanning Electron Microscopy (SEM)

Samples of gold nanomaterial generated in the VFD were added to a silicon substrate as a colloidal suspension in Milli‐Q water by drop casting followed by evaporation under ambient conditions. Morphologies, size, and shapes of the particles were studied using a scanning electron microscope (SEM, FEI F50) operating with an accelerating voltage of 10 kV with a 10 mm working distance.

##### Characterizations: Transmission Electron Microscopy (TEM)

TEM was performed using a JEOL JEM‐F200 Multi‐Purpose FEG‐S/TEM operating at an accelerating voltage of 200 kV. Image J was used for processing the images.

##### Characterizations: Thermal Gravitational Analysis (TGA)

Under nitrogen was recorded for gold nanosheets between 100 and 1000 °C.

##### Characterizations: Raman Spectroscopy

Raman spectra were acquired using a Witec alpha300R Raman microscope at an excitation laser wavelength of 532 nm with a 40X objective (numerical aperture 0.60). Integration times for single spectra were typically 20 s and averaged from 1 to 3 accumulations. The grating used was 600 g mm^−1^ which gives a spectral resolution of ≈3 to 4 wavenumbers.

##### Characterizations: Magnetic Force Microscopy Measurement (MFM)

All magnetic force microscopy (MFM) images and measurements were carried out using a Bruker ICON head at room temperature using magnetized silicon cantilevers coated with a magnetic cobalt chromium, CoCr, (MESP‐V2 from Bruker) with a normal resonance frequency of 75 kHz and normal spring constant of 3 N m^−1^. The coating produced a coercivity of ≈400 Oe.

##### Characterizations: Electron Paramagnetic Resonance (EPR)

Continuous‐wave (CW) X‐band (9.6139 GHz GHz) EPR spectra were recorded on a Bruker Elexsys E500 spectrometer equipped with an ElexSys Super High Sensitivity Probehead and He cooling (Bruker waveguide Cryogen‐free system with recirculator). The magnetic field was calibrated with 2,2‐diphenyl‐1‐picrylhydrazyl (*g* = 2.0036) and measurements were carried out at 10 K using a modulation amplitude of 0.7 mT, a modulation frequency of 100 kHz and a microwave power of 0.126 mW. Simulations were carried out with EasySpinn.^[^ ^48^ ^]^

##### Characterizations: Computational Methods

Self‐consistent calculations in VASP run reasonably fast because only the valence electrons are treated explicitly, taking electron–core interactions into account using the projector augmented wave method.^[^ ^49^, ^50^ ^]^ All calculations include spin polarization and use the generalized gradient approximation (GGA) of Perdew, Burke, and Ernzerhof (PBE)^[^ ^51^ ^]^ for the exchange‐correlation functional. Convergence tests show that a plane‐wave energy cut‐off of 500 eV optimizes computing speed and numerical accuracy. The optimum lattice constant of gold was found to be 4.158 Å. Electronic wave functions are expanded in this DFT package using basis sets of plane‐waves, and assume that the system of atoms under investigation is periodic in three dimensions. Modeling gold surfaces was therefore carried out using supercells large enough to maintain a separation between neighboring images of about 30 Å. Slabs that are seven‐atomic layers thick were used to simulate the pristine (111) surface of gold. Atomic positions were relaxed until the magnitude of forces on all atoms fell below 0.01 eV Å^−1^. Different starting magnetizations were used in an unbiased search for electronic ground states.

##### Characterizations: Electrochemistry and Electrode Preparation

Autolab potentiostat PGSTAT204 (Metrohm, Switzerland) was used for all the electrochemistry measurements. Glassy carbon electrode (CHI104, 3 mm diameter), platinum wire electrode (CHI115), aqueous Ag/AgCl reference electrode (CHI111, with porous Teflon tip), and three‐electrode electrochemical cell stand (CHI220) were purchased from CH Instruments, Inc. (Texas, USA). The glassy carbon electrodes were polished by alumina slurry (0.05/0.3/1.0 μm, Ionode) for minimum 10 min prior to use, and the platinum wire counter electrode was flame‐cleaned by gas torch. As for aqueous Ag/AgCl reference electrode, it was filled with fresh 1 M KCl solution before a series of electrochemical test in 0.5 M H_2_SO_4_. Milli‐Q water (TOC 4 ppm, 18.2 ΩM · cm@ 25 °C) and ethanol were used for the dilution of AuNPs dispersions. The supporting electrolyte was 0.5 M H_2_SO_4_ (10 mL), which was replaced every 10 scans, voltage Range (vs RHE) −0.6 to 0.235 V. A drop (5 μL) of diluted gold sample was dispersion onto glassy carbon electrode followed by drying in vacuo for 30 min, with then the addition of a drop (10 μL) of diluted Nafion solution (in EtOH) on the glassy carbon electrode for protection of the metal sample followed drying also in vacuo. All electrochemistry studies included magnetic stirring at 1200 rpm, with the cyclic voltammetry (CV) over the voltage range +0.335 to −0.765 V for 10 scans.

## Conflict of Interest

The authors declare no conflict of interest.

## Author Contributions

**Badriah M. Alotaibi**: conceptualization (equal); formal analysis (lead); investigation (equal); methodology (equal); validation (equal); writing—original draft (equal); writing—review amp; editing (equal). **Soraya Rapheima:** data curation (equal); formal analysis (equal). **Po‐Wei Yu**: data curation (equal); formal analysis (equal); writing—original draft (equal). **Xianjue Chen**: formal analysis (equal); writing—original draft (supporting); writing—review amp; editing (supporting). **Tanglaw Roman**: formal analysis (equal); methodology (equal); writing—original draft (equal); writing—review amp editing (supporting). **Christopher T. Gibson**: formal analysis (supporting); writing—review & editing (supporting). **Tiexin Lib**: formal analysis (supporting); writing—review & editing (supporting). **Dechao Chen**: formal analysis (supporting); writing—review & editing (supporting). **Elsa Antunes**: investigation (supporting) writing—original draft (supporting); writing—review & editing (supporting). **Qin Li**: formal analysis (supporting); writing—original draft (supporting); writing—review & editing(supporting). **Mats R. Anderson**: investigation (supporting); writing—original draft (supporting); writing—review & editing (supporting). **Nadim Darwish**: formal analysis (equal); investigation (supporting); methodology (equal); writing—original draft (equal); writing—review & editing (equal). **Colin L. Raston**: conceptualization (equal); data curation(supporting); formal analysis(equal) funding acquisition (lead); investigation (lead); methodology (equal) project administration (lead); resources (lead); supervision (lead); validation (equal); visualization (equal); writing—original draft (equal); writing—review & editing (lead)

## Supporting information

Supplementary Material

## Data Availability

The data that support the findings of this study are available in the Supporting Information of this article.

## References

[1] S. Singh , S. Jain , P. S. Venkateswaran , A. K. Tiwari , M. R. Nouni , J. K. Pandey , S. Goel , Renewable Sustainable Energy Rev. 2015, 51, 623.

[2] Y. Tong , J. Mater. Chem. A 2024, 12, 3844.

[3] A. Kazemi , F. Manteghi , Z. Tehrani , ACS Omega 2024, 9, 7310. 38405471 10.1021/acsomega.3c07911 PMC10882616

[4] S. Perumal , I. Pokhrel , U. Muhammad , X. Shao , Y. Han , M. Kim , H. Lee , ACS Mater. Lett. 2024, 6, 3625.

[5] Y. Y. Bai , Electroanalysis 2024, 36, e202300354.

[6] T.‐W. Chen , S. M. Chen , P. Kalimuthu , G. Anushya , R. Kannan , A. G. Al‐Sehemi , M. Vinitha , A. Saranvignesh , M. A. Mohammed , J. Suganya , C. M. Thavasimuthu , R. Ramachandran , Int. J. Electrochem. Sci. 2024, 19, 100576.

[7] M. O. Stetsenko , S. P. Rudenko , L. S. Maksimenko , B. K. Serdega , O. Pluchery , S. V. Snegir , Nanoscale Res. Lett. 2017, 12, 1. 28499336 10.1186/s11671-017-2107-8 PMC5427009

[8] I. Hammami , N. M. Alabdallah , J. King Saud Univ. Sci. 2021, 33, 101560.

[9] A. Hernando , P. Crespo , M. A. Garcia , M. Coey , A. Ayuela , P. M. Echenique , Phys. Status Solidi B 2011, 248, 2352.

[10] A. Hernando , P. Crespo , M. Garcia , Phys. Rev. Lett. 2006. 96, 057206. 16486977 10.1103/PhysRevLett.96.057206

[11] R. Jin , C. Zeng , M. Zhou , Y. Chen , Chem. Rev. 2016, 116, 10346. 27585252 10.1021/acs.chemrev.5b00703

[12] C. M. Wu , C. Y. Li , Y. T. Kuo , C. W. Wang , S. Y. Wu , W. H. Li , J. Nanopart. Res. 2010, 12, 177.

[13] G. L. Nealon , B. Donnio , R. Greget , J. P. Kappler , E. Terazzi , J. L. Gallani , Nanoscale 2012, 4, 5244. 22814797 10.1039/c2nr30640a

[14] M. Ramchandani , J. Phys. C: Solid State Phys. 1970, 3, S1.

[15] S. Trudel , Gold Bull. 2011, 44, 3.

[16] V. Amendola , M. Meneghetti , M. Stener , Y. Guo , S. Chen , P. Crespo , A. G. Miguel , H. Antonio , P. Paolo , L. Pasquato , Comprehensive Analytical Chemistry, Elsevier, Amsterdam 2014, pp. 81–152.

[17] C. Y. Li , S. K. Karna , C. W. Wang , W. H. Li , Int. J. Mol. Sci. 2013, 14, 17618. 23989607 10.3390/ijms140917618 PMC3794745

[18] V. Tuboltsev , A. Savin , A. Pirojenko , J. Raisanen , ACS Nano 2013, 7, 6691. 23829643 10.1021/nn401914b

[19] M. Agrachev , S. Antonello , T. Dainese , M. Ruzzi , A. Zoleo , E. Aprà , G. Niranjan , F. Alessandro , S. Luca , F. Maran , ACS Omega 2017, 2, 2607. 31457603 10.1021/acsomega.7b00472 PMC6640951

[20] U. Maitra , B. Das , N. Kumar , A. Sundaresan , C. N. R. Rao , Chem. Phys. Chem. 2011, 12, 2322. 21744458 10.1002/cphc.201100121

[21] A. Hernando , P. Crespo , M. A. García , E. F. Pinel , J. De La Venta , A. Fernández , S. Penadés , Phys. Rev. B—Condens. Matter. Mater. Phys. 2006, 74, 052403.

[22] K. S. Krishna , P. Tarakeshwar , V. Mujica , C. S. Kumar , Small 2014, 10, 907. 24150895 10.1002/smll.201302393

[23] J. de la Venta , A. Pucci , E. Fernández Pinel , M. A. García , C. de Julián Fernandez , P. Crespo , P. Mazzoldi , G. Ruggeri , A. Hernando , Adv. Mater. 2007, 19, 875.

[24] P. Dong , E. A. Fisher , M. V. Meli , S. Trudel , Nanoscale 2020, 12, 19797. 32966519 10.1039/d0nr05674j

[25] S. Harke , A. Habibpourmoghadam , A. B. Evlyukhin , A. Calà Lesina , B. N. Chichkov , Sci. Rep. 2023, 13, 21588. 38062118 10.1038/s41598-023-48813-y PMC10703919

[26] K. Vimalanathan , X. Chen , C. L. Raston , Chem. Commun. 2014, 50, 11295. 10.1039/c4cc03126a 24918519

[27] L. Yasmin , X. Chen , K. A. Stubbs , C. L. Raston, Sci. Rep. 2013, 3, 2282. 23884385 10.1038/srep02282 PMC3722563

[28] J. Britton , K. A. Stubbs , G. A. Weiss , C. L. Raston , Chem.–A Eur. J. 2017, 23, 13270. 10.1002/chem.201700888 PMC6014698 28597512

[29] B. M. Alotaibi , Z. Gardner , K. Vimalanathan , X. Chen , T. M. Alharbi , C. L. Raston , Small Sci. 2024, 4, 2300312. 40212757 10.1002/smsc.202300312 PMC11935251

[30] H. Li , X. Qi , J. Wu , Z. Zeng , J. Wei , H. Zhang , ACS Nano 2013, 7, 2842. 23442061 10.1021/nn400443u

[31] V. T. Tran , H. Zhou , S. Lee , S. C. Hong , J. Kim , S. Y. Jeong , J. Lee , ACS Appl. Mater. Interfaces 2015, 7, 8650. 25856000 10.1021/acsami.5b00904

[32] S. Yang , C. Wang , H. Sahin , H. Chen , Y. Li , S. S. Li , A. Suslu , F. M. Peeters , Q. Liu , J. Li , S. Tongay , Nano Lett. 2015, 15, 1660. 25642738 10.1021/nl504276u

[33] A. Wadas , R. Wiesendanger , P. Novotny , J. Appl. Phys. 1995, 78, 6324.

[34] T. Shinjo , T. Okuno , R. Hassdorf , K. Shigeto , T. Ono , Science 2000, 289, 930. 10937991 10.1126/science.289.5481.930

[35] P. Milde , D. Köhler , J. Seidel , L. M. Eng , A. Bauer , A. Chacon , J. Kindervater , S. Mühlbauer , C. Pfleiderer , S. Buhrandt , C. Schütte , A. Rosch , Science 2013, 340, 1076. 23723232 10.1126/science.1234657

[36] I. Kézsmárki , S. Bordács , P. Milde , E. Neuber , L. M. Eng , J. S. White , H. M. Rønnow , C. D. Dewhurst , M. Mochizuki , K. Yanai , H. Nakamura , D. Ehlers , V. Tsurkan , A. Loidl , Nat. Mater. 2015, 14, 1116. 26343913 10.1038/nmat4402

[37] Z. Wang , A. Berbille , Y. Feng , S. Li , L. Zhu , W. Tang , Z. L. Wang , Nat. Commun. 2022, 13, 130. 35013271 10.1038/s41467-021-27789-1 PMC8748705

[38] Z. L. Wang , Rep. Prog. Phys. 2021, 84, 096502.

[39] C. S. Neves , P. Quaresma , P. V. Baptista , P. A. Carvalho , J. P. Araújo , E. Pereira , P. Eaton , Nanotechnology 2010, 21, 305706. 20610872 10.1088/0957-4484/21/30/305706

[40] A. Schwarz , R. Wiesendanger , Nano Today 2008, 3, 28.

[41] Y. Feng , P. M. Vaghefi , S. Vranjkovic , M. Penedo , P. Kappenberger , J. Schwenk , X. Zhao , A. O. Mandru , H. J. Hug , J. Magn. Magn. Mater. 2022, 551, 169073.

[42] Y. Inagaki , H. Yonemura , N. Sakai , Y. Makihara , T. Kawae , S. Yamada , Appl. Phys. Lett. 2016, 109, 072404.

[43] M. Agrachev , M. Ruzzi , A. Venzo , F. Maran , Acc. Chem. Res. 2018, 52, 44. 30480998 10.1021/acs.accounts.8b00495

[44] A. Hernando , P. Crespo , M. Garcia , Phys. Rev. Lett. 2006, 96, 057206. 16486977 10.1103/PhysRevLett.96.057206

[45] A. Hernando , P. Crespo , M. A. Garcia , M. Coey , A. Ayuela , P. M. Echenique , Phys. Status Solidi B 2011, 248, 2352.

[46] M. Mainak , J. A. Eukel , J. Eukel , W. J. YL , B. Natnael , L. Tzu‐Yu , M. Francesca , J. Phys. Chem. B 2012, 116, 6536.22587569

[47] K. L. Knoche Gupta , H. C. Lee , J. Leddy , ACS Phys. Chem. Au 2024, 4, 148. 38560752 10.1021/acsphyschemau.3c00039 PMC10979484

[48] S. Stoll , A. Schweiger , J. Magn. Reson. 2006, 178, 42. 16188474 10.1016/j.jmr.2005.08.013

[49] P. E. Blöchl , O. Jepsen , O. K. Andersen , Phys. Rev. B 1994, 49, 16223. 10.1103/physrevb.49.16223 10010769

[50] G. Kresse , D. Joubert , Phys. Rev. B 1999, 59, 1758.

[51] J. P. Perdew , K. Burke , M. Ernzerhof , Phys. Rev. Lett. 1996, 77, 3865. 10062328 10.1103/PhysRevLett.77.3865

