# Supplementary material for: Vortex Fluidic Mediated Synthesis of Enhanced Hydrogen Producing Magnetic Gold
Source: Small Sci. 2025 Feb 3;5(4):2400449. doi: 10.1002/smsc.202400449 (PMC12244516; doi:10.1002/smsc.202400449)
Supplement: Supplementary file 1 — Supplementary Material [file SMSC-5-2400449-s001.pdf]

Supplementary Information

Vortex Fluidic Mediated Synthesis of Enhanced Hydrogen Producing Magnetic Gold

Badriah M. Alotaibi,[a] Soraya Rapheima,[b] Po-Wei Yu,[a] Xianjue Chen,[c] Tanglaw Roman[a,d] Christopher T. Gibson,[a,e] Tiexin Lib,[b] Dechao Chen,[f] Elsa Antunes[g], Qin Li,[f] Nadim Darwish,[b] Mats R. Anderson,[a] Colin L. Raston[a*]

[a] Flinders Institute for Nanoscale Science and Technology, College of Science and Engineering, Flinders University, Adelaide, SA 5042, Australia.

[b] School of Molecular and Life Sciences, Curtin Institute of Functional Molecules and Interfaces, Curtin University, Bentley, WA 6102, Australia.

[c] School of Environmental and Life Sciences, The University of Newcastle, Callaghan, New South Wales 2308, Australia

[d] Flinders Microscopy and Microanalysis, College of Science and Engineering, Flinders University, Bedford Park, SA 5042, Australia

[e] Adelaide Microscopy, The University of Adelaide, Adelaide, South Australia 5000, Australia

[f] Queensland Micro- and Nanotechnology Centre School of Engineering and Built Environment, Griffith University, Nathan , QLD 4111

[g] College of Science and Engineering James Cook University Townsville, Queensland 4811, Australia

Address for correspondence: colin.raston@flinders.edu.au

Abstract

While bulk gold is well known to be diamagnetic, there is growing experimental and theoretical work supporting the formation of nano gold with unconventional magnetic properties. However, access to such magnetic gold nanoparticles at scale is limited. We have established that magnetic gold particles are readily accessible when exposing aqueous solutions of auric acid ($H[AuCl_4]$) to UV irradiation (λ = 254 nm) under high shear in a vortex fluidic device (VFD), as a photo-contact electrification process. Thin films of liquid in the VFD down to ~200 μm thick are generated in a tilted rapidly rotating angled glass tube with induced mechanical energy imparted under high shear, which when exposed to UV, reduces Au^{3+} to elemental gold without the need for adding reducing agents, unlike in the conventional synthesis of nanogold particles. We report the use of magnetic force microscopy (MFM) to show that VFD generated 2D gold sheets have magnetic gold nanoparticles embedded in them, with the material electron paramagnetic resonance (EPR) active. We also report theoretical insights into the origin of the magnetism and that the material shows a dramatic enhancement of catalytic activity in the hydrogen generation reaction relative to using traditionally produced gold nanoparticles of comparable size.

Table of Contents

Vortex Fluidic Mediated Synthesis of Enhanced Hydrogen Producing Magnetic Gold 1

AFM topography and MFM images, and MFM response for gold sheets 2

The MFM measurements correspond to the VFD-fabricated gold with xerogel silica 3

AFM topography and MFM images, and MFM response for gold formed in the VFD under nitrogen atmosphere. 5

TEM Study.. 6

MFM measurements of pre-papered gold nanoparticles (using reduction of $HAuCl_4$ to metallic gold by ascorbic acid) before VFD processing. 6

MFM measurements of pre-papered gold nanoparticles (using reduction of $HAuCl_4$ to metallic gold by ascorbic acid) After VFD processing.. 8

More analytical techniques study on gold surface.. 9

The calculated mass activity of magnetic gold in comparison with classic gold and gold disk. 11

AFM topography and MFM images, and MFM response for gold sheets

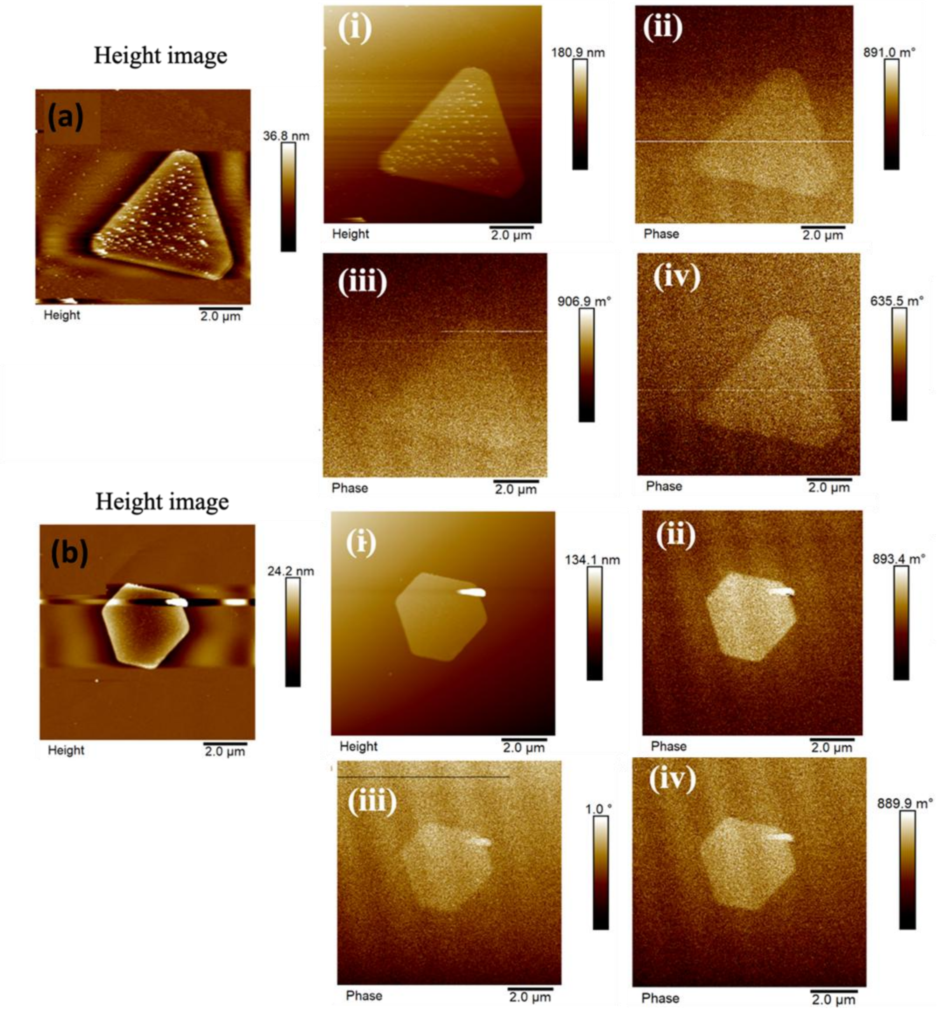

Figure S1. (a) AFM topography for 2D triangular gold sheets with an array of AuNPs, (i) MFM phase image and the corresponding phase shift profile. (b) AFM topography image for a 2D hexagonal gold sheets, (i) MFM phase image and the corresponding phase shift profile. The lift scan height is 30-50-100 nm in (ii-iii-iv) respectively. VFD processing was as follows: ω = 5k rpm, θ = 45˚, λ = 254 nm, confined mode, c = 3.7 mM, t = 60 min, under air atmosphere.

The MFM measurements correspond to the VFD-fabricated gold with xerogel silica.

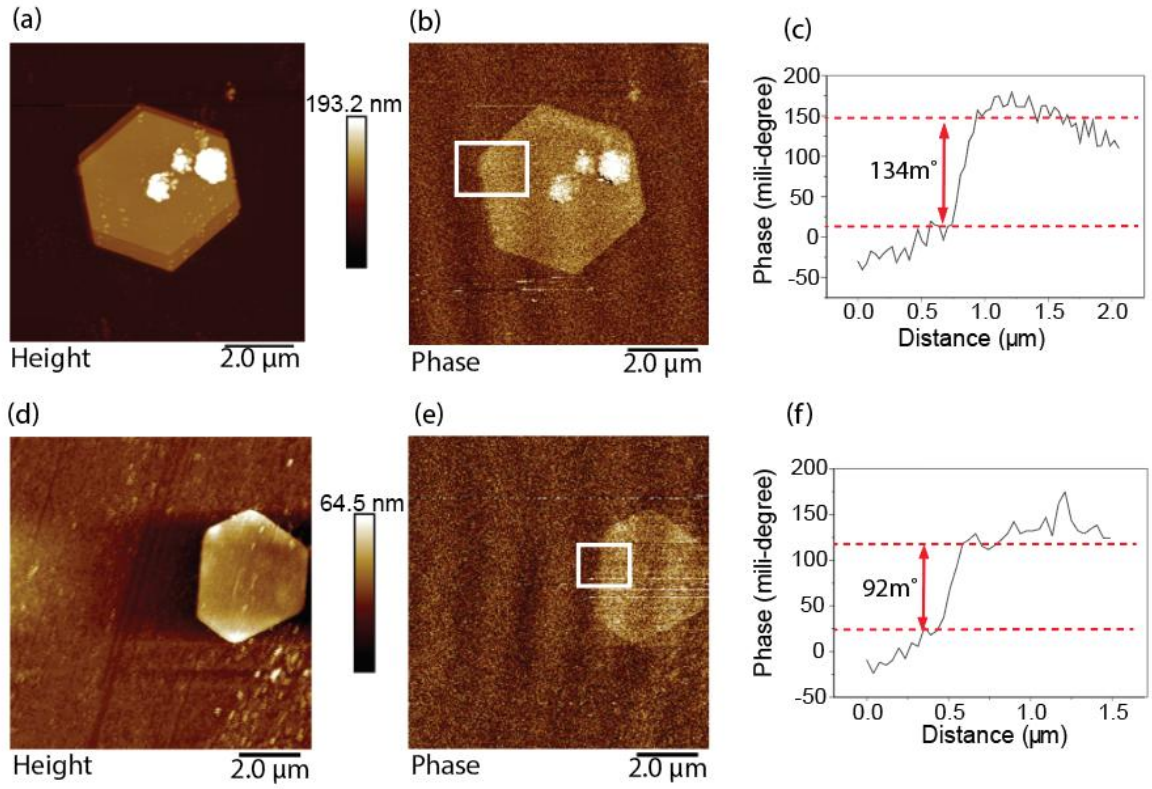

Figure S2. (a) MFM topography, (b) MFM phase image and (c) the corresponding phase shift profile of the rectangle in b, respectively. (d) MFM topography image, (e) MFM phase image and (f) the corresponding phase shift profile of the rectangle in e, respectively. The lift scan height is 50 nm. The MFM measurements correspond to the VFD-fabricated gold with xerogel silica. The VFD processing: ω = 5k rpm, concentration of auric acid = 3.7 mM and 45°, UV light for 60 min processing under air atmosphere.

AFM topography and MFM images, and MFM response for gold formed in the VFD under nitrogen atmosphere.

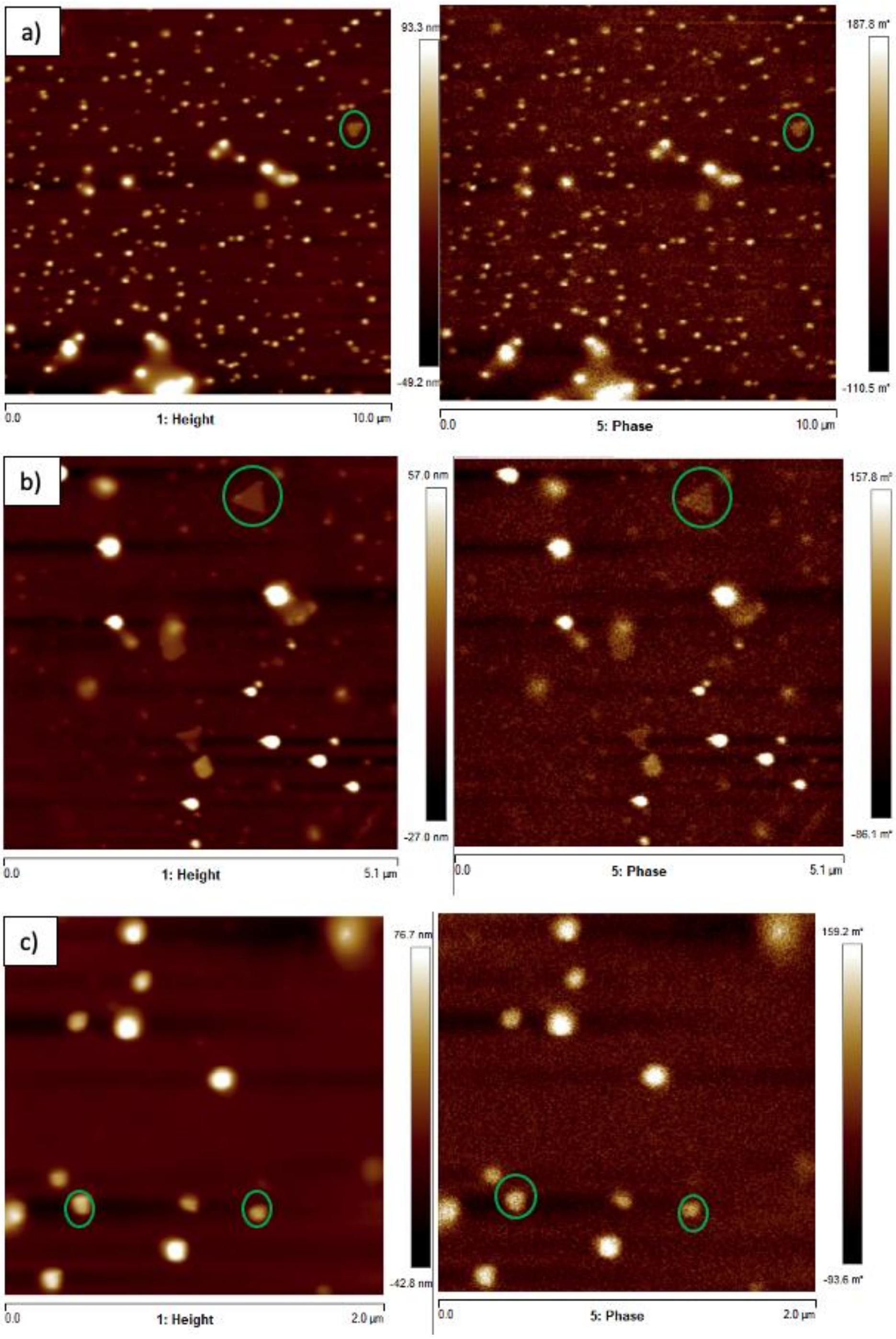

Figure S3. AFM topography and MFM images, and MFM response for gold formed under nitrogen atmosphere. (A-C) AFM topography image, MFM phase image for the left scan height is 50 nm and MFM phase image for the lift scan height is 100 nm with all the corresponding phase shift profile, prepared at (ω = 5k rpm, concentration of auric acid = 3.7 mM and 45°, UV light for 60 min processing under N_2 atmosphere).

TEM Study

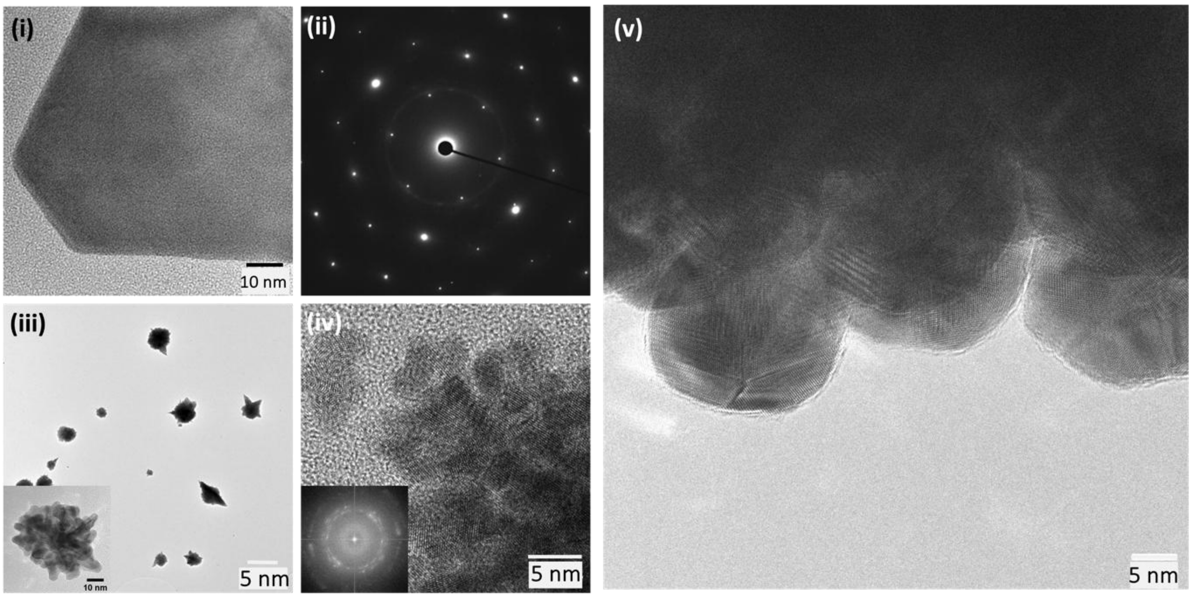

Figure S4: (i) TEM image of a gold nanosheet and (ii) the corresponding selected area electron diffraction pattern. (iii-v) TEM image of gold clusters with a typical cluster shown in the set and a zoomed-in TEM image showing the lattice fringes of the AuNPs in a cluster, with the inset showing the FFT pattern generated from the area shown in (iv). (iv-v) HRTEM image showing the lattice of the AuNPs in a cluster.

MFM measurements of pre-papered gold nanoparticles (using reduction of $HAuCl_4$ to metallic gold by ascorbic acid) before VFD processing.

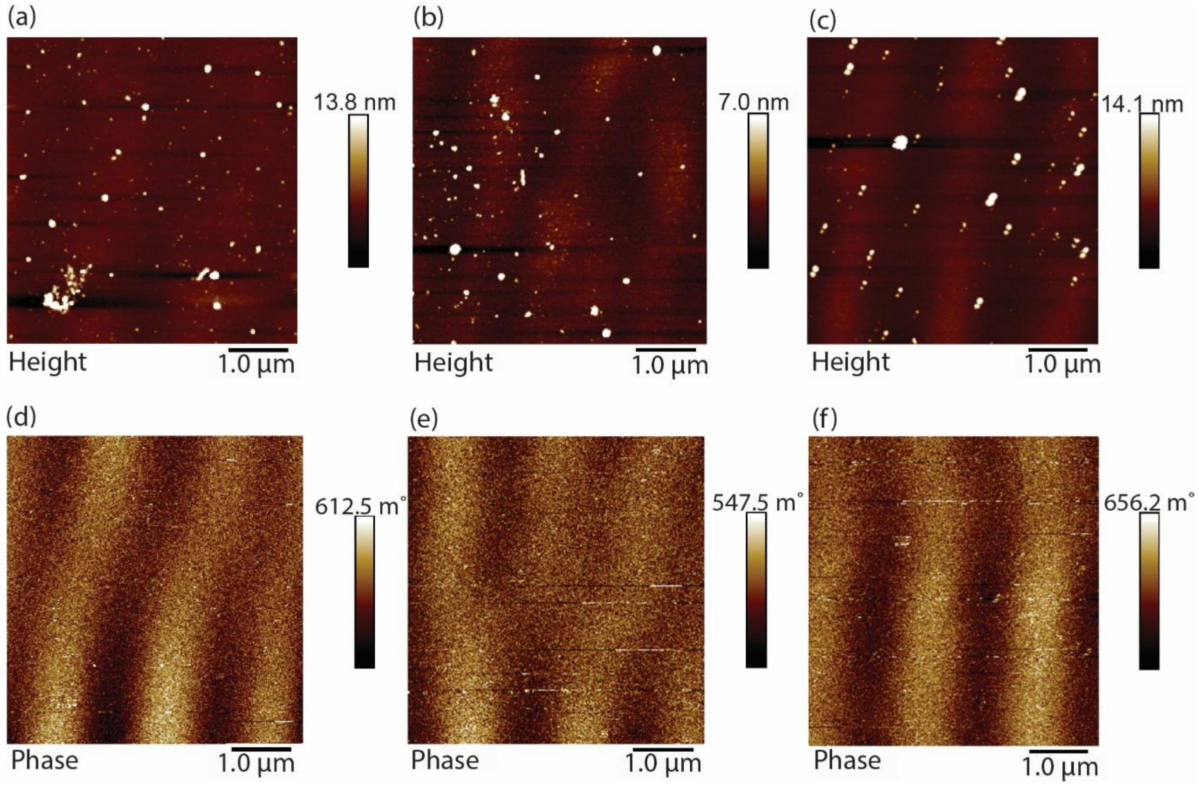

Figure S5. MFM measurements of pre-papered gold nanoparticles (using reduction of $HAuCl_4$ to metallic gold by ascorbic acid) before VFD processing. (a–c) MFM height topography images. (d–f) MFM phase images showing no magnetic response. The lift scan height is 50 nm. The nanoparticles with ascorbic acid were prepared by mixing 1:1 ascorbic acid solution (concentration of AA, 1 mg in 1 mL of water), with 3.7 mM of gold chloride solution and stir for 10 min.

MFM measurements of pre-papered gold nanoparticles (using reduction of $HAuCl_4$ to metallic gold by ascorbic acid) After VFD processing

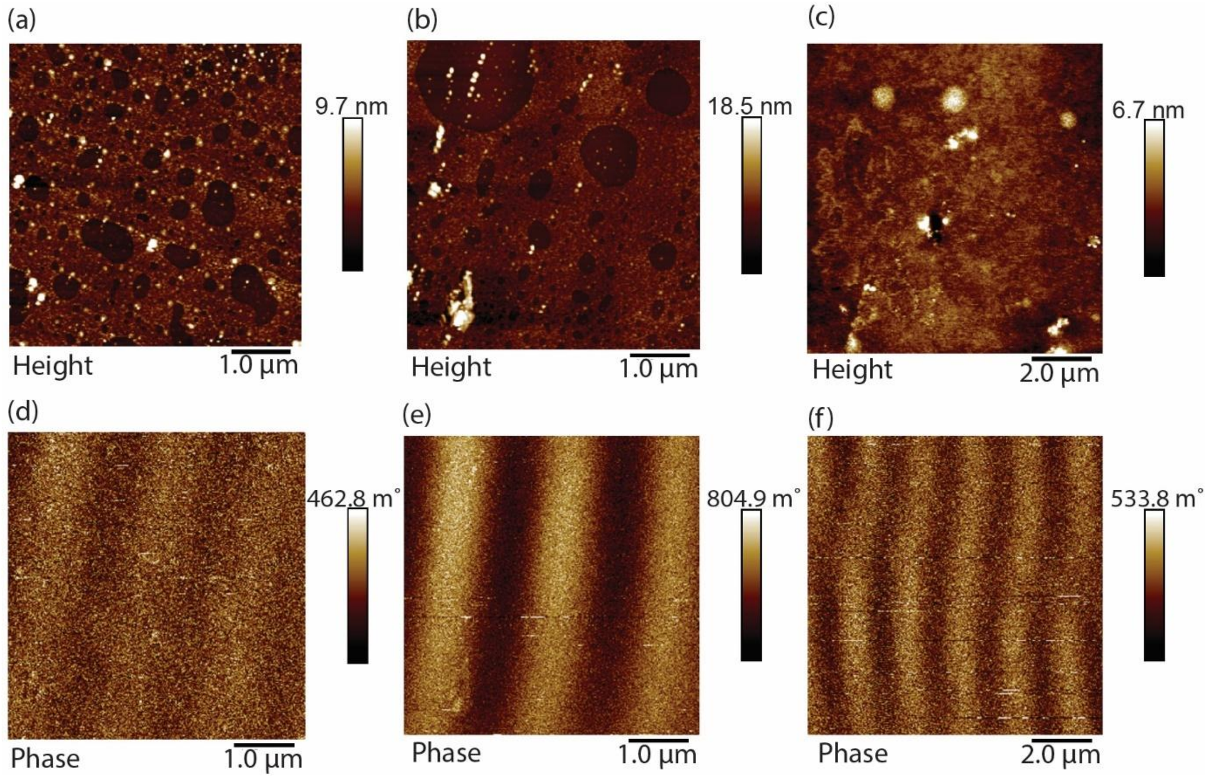

Figure S6. MFM measurements on pre-papered gold nanoparticles (using reduction of $HAuCl_4$ to metallic gold by ascorbic acid), processed in the VFD. (a–c) MFM height topography images. (d–f) MFM phase images showing no magnetic response. The lift scan height is 30 nm in (d) and 50 nm in (e and f). The nanoparticles with ascorbic acid were prepared by mixing 1:1 ascorbic acid solution (concentration of AA, 1 mg in 1 mL of water), with 3.7 mM of gold chloride solution and the VFD processing as follow: ω = 5k rpm, 60 min, UV light, under air atmosphere

More analytical techniques study on gold surface

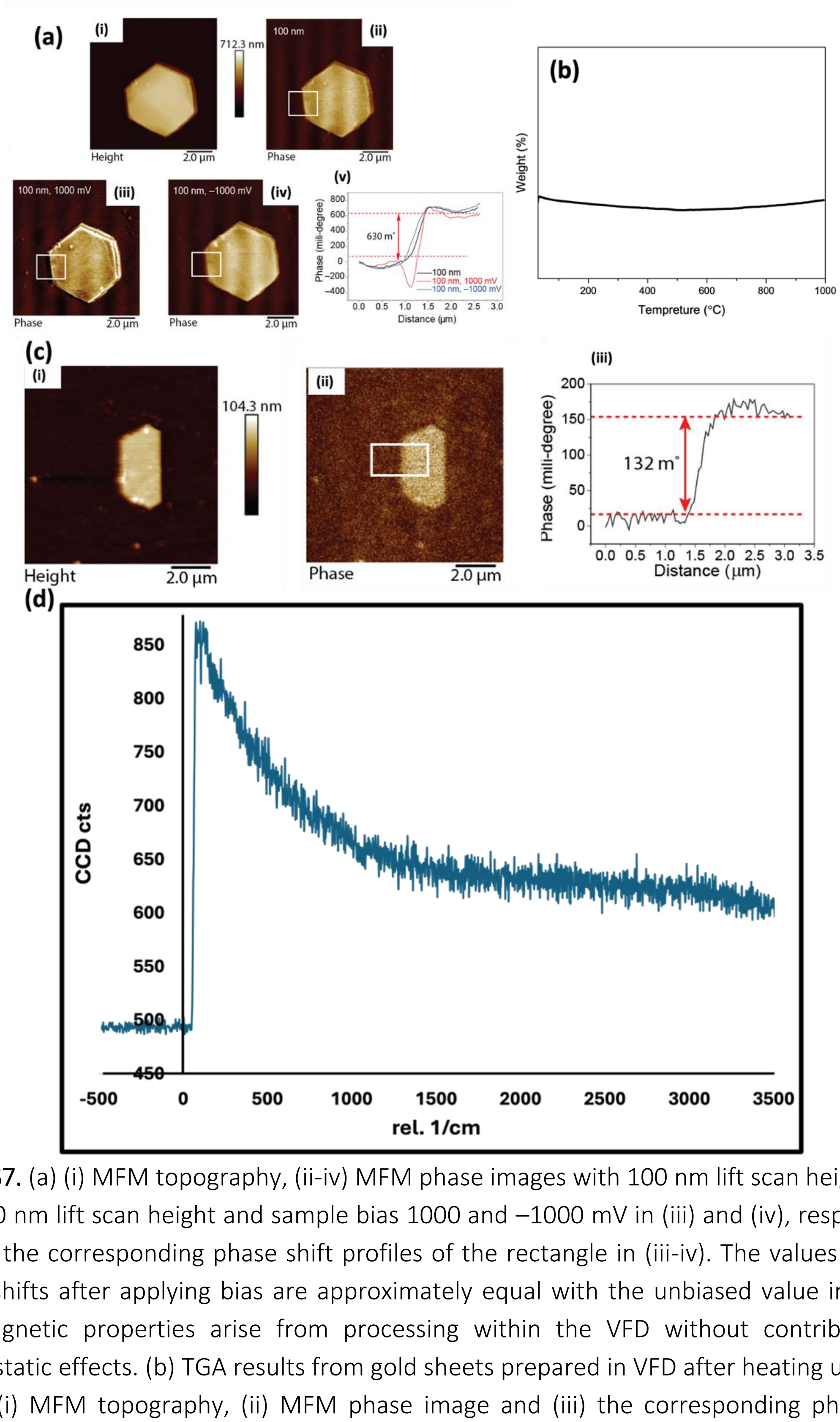

Figure S7. (a) (i) MFM topography, (ii-iv) MFM phase images with 100 nm lift scan height in (ii) and 100 nm lift scan height and sample bias 1000 and –1000 mV in (iii) and (iv), respectively, and (v) the corresponding phase shift profiles of the rectangle in (iii-iv). The values of MFM phase shifts after applying bias are approximately equal with the unbiased value indicating the magnetic properties arise from processing within the VFD without contribution of electrostatic effects. (b) TGA results from gold sheets prepared in VFD after heating up to 900 ˚C, (c) (i) MFM topography, (ii) MFM phase image and (iii) the corresponding phase shift profile of the rectangle in (ii), respectively. The lift scan height is 50 nm. The MFM response corresponds to the fabricated gold sheet within the VFD after heating up to 900 ˚C indicating

no changes in gold sheets after heating. (d) Raman Spectra for gold nanosheets. VFD processing was as follows: ω = 5k rpm, θ = 45˚, λ = 254 nm, t = 60 min, confined mode, c = 3.7 mM, air atmosphere.

The current density of gold nanoparticles with different concentrations (in H_2O) with the HER activity in 0.5 M H_2SO_4 from 0.2mM gold NPs dispersed in 50 vol% ethanol/water, and (b) shows the calculated mass activity.

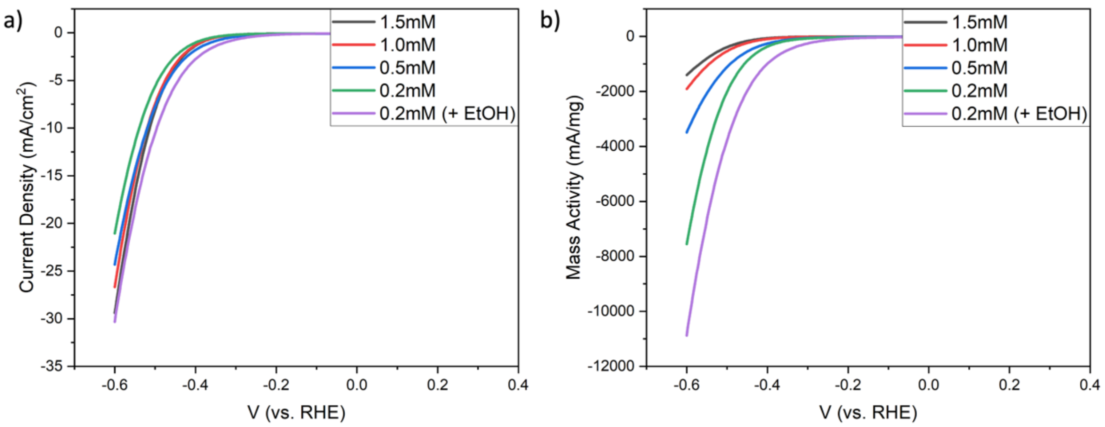

Figure S8. (a) Current density of gold nanoparticles with different concentrations (in H_2O) with the HER activity in 0.5 M H2SO4 from 0.2 mM gold NPs dispersed in 50 vol% ethanol/water, and (b) showing the calculated mass activity.

The calculated mass activity of magnetic gold in comparison with classic gold and gold disk.

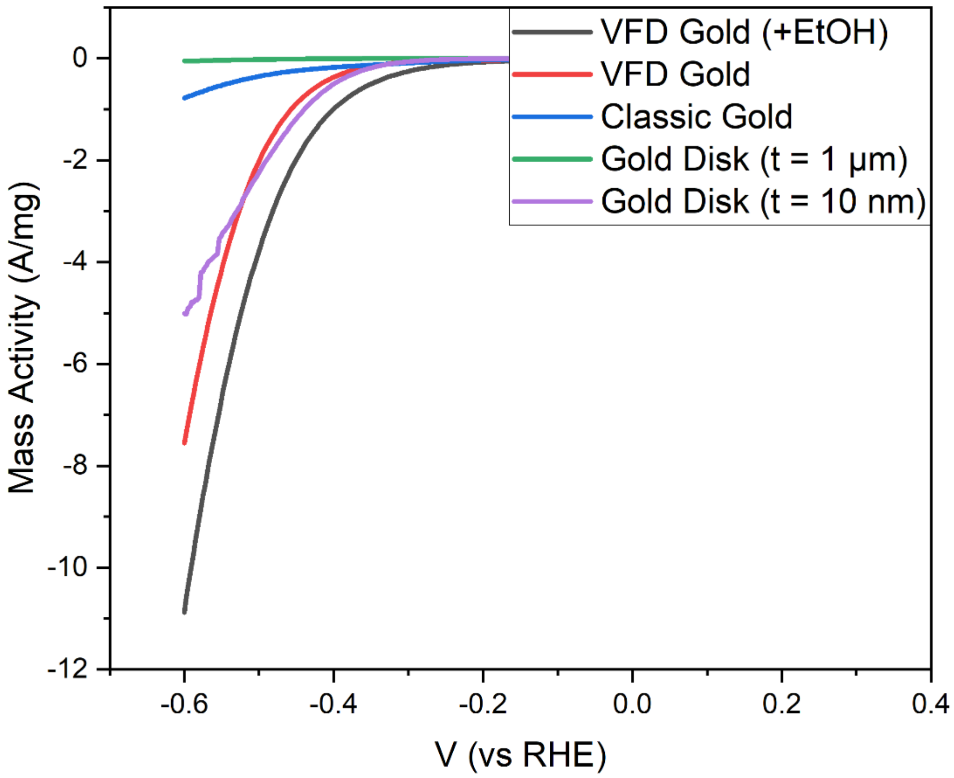

Figure S9. The calculated mass activity comparison between magnetic gold in water and ethanol in comparison with classic gold and gold disk.
